# Quercetin Reverses Rat Liver Preneoplastic Lesions Induced by Chemical Carcinogenesis

**DOI:** 10.1155/2017/4674918

**Published:** 2017-06-27

**Authors:** Gabriela Carrasco-Torres, Hugo Christian Monroy-Ramírez, Arturo Axayacatl Martínez-Guerra, Rafael Baltiérrez-Hoyos, María de los Ángeles Romero-Tlalolini, Saúl Villa-Treviño, Xariss Sánchez-Chino, Verónica Rocío Vásquez-Garzón

**Affiliations:** ^1^Departamento de Biología Celular, Centro de Investigación y de Estudios Avanzados del Instituto Politécnico Nacional, Ciudad de México, Mexico; ^2^Departamento de Farmacología, Facultad de Medicina Mexicali, Universidad Autónoma de Baja California, Baja California, Mixicali, BC, Mexico; ^3^CONACYT, Facultad de Medicina y Cirugía, Universidad Autónoma Benito Juárez de Oaxaca, Oaxaca de Juárez, OAX, Mexico; ^4^Dirección de Investigación y Desarrollo Tecnológico, Universidad Politécnica Mesoamericana, Tenosique, TAB, Mexico

## Abstract

Quercetin is a flavonoid widely studied as a chemopreventive agent in different types of cancer. Previously, we reported that quercetin has a chemopreventive effect on the liver-induced preneoplastic lesions in rats. Here, we evaluated if quercetin was able not only to prevent but also to reverse rat liver preneoplastic lesions. We used the modified resistant hepatocyte model (MRHM) to evaluate this possibility. Treatment with quercetin was used 15 days after the induction of preneoplastic lesions. We found that quercetin reverses the number of preneoplastic lesions and their areas. Our results showed that quercetin downregulates the expression of EGFR and modulates this signaling pathway in spite of the activated status of EGFR as detected by the upregulation of this receptor, with respect to that observed in control rats. Besides, quercetin affects the phosphorylation status of Src-1, STAT5, and Sp-1. The better status of the liver after the treatment with quercetin could also be confirmed by the recovery in the expression of IGF-1. In conclusion, we suggest that quercetin reversed preneoplastic lesions by EGFR modulation and the activation state of Src, STAT5, and Sp1, so as the basal IGF-1.

## 1. Introduction

Hepatocellular carcinoma (HCC) is one of the most common and deadly cancers worldwide [1]. HCC, as another type of cancer, is established by a multistep and multifactorial process; there, we can distinguish three stages, the initiation, the promotion, and the progression. Different risk factors stimulate the progression of the malignancy, and when it is detected, actual treatments are not efficient. Some of these factors are the infection with hepatitis B or C virus, heavy alcohol intake, nonalcoholic steatohepatitis, and exposure to toxic substances such as aflatoxin B or vinyl chloride, among others. Even if the relation is not clear, the diet has been also shown to play an important role in the development of HCC [2, 3].

Antioxidants in the diet, such as flavonoids contained in several fruits and vegetables, have a beneficial effect on liver tumors in animal models and induce apoptosis in cancer cell lines [2, 4, 5]. Quercetin, a flavonoid widely studied as a chemopreventive agent in different types of cancer, is considered an excellent antioxidant with a proapoptotic effect and able to inhibit the growth of different cancer cell lines [6]. It has been proved that quercetin is able to inhibit the metabolic activity and cell death by apoptosis in HCC cell lines like HepG2, HuH7, and Hep3B2 [7]. It also induces a decrease in oxidative stress and a significant decrease of antioxidant activity in the liver of rats treated with N-nitrosodiethylamine as a cancer inducer [5]. We have also previously reported that quercetin has a chemopreventive effect on the liver of rats when this is used previously to the use of the MRHM to induce HCC. Quercetin reduces the number of cells initiated to cancer through decreasing lipoperoxidation, activating caspases 3 and 9, and promoting the enzymatic and nonenzymatic antioxidant defense system during the initiation of hepatocarcinogenesis [8, 9]. While the use of chemoprotective substances would have a major impact on the incidence of HCC, it is also necessary to discover substances that contribute to reverse the injuries, since this type of cancer is usually detected in late stages.

The MRHM is a well-characterized model useful for the analysis and quantitation of different stages of HCC. On this model, N-diethylnitrosamine (DEN) in single doses is applied as an initiator agent. Later, 2-acetylaminofluorene (2-AAF) and partial hepatectomy act in promoting the development of initiated cells and their progression to altered hepatic foci [10, 11]. Moreover, the detection of gamma-glutamyl transpeptidase (GGT) is a useful tool, which has been used in detecting HCC-related lesions. GGT is not present in hepatocytes of adult rats, but it is detected in altered hepatic foci induced by carcinogens, in most animal models, from the initial lesions caused by carcinogens until the tumor formation [9].

It has been proposed that quercetin can influence the activation of epidermal growth factor receptor (EGFR) through the overexpression of some ligands like amphiregulin [12] and also has been shown that quercetin, at a dose of 10 *μ*M, decreases the phosphorylation of EGFR in prostate cancer cells (PC3), producing a diminished expression of target proteins like vimentin, N-cadherin, and cyclin D1 [13]. Quercetin has been also proven to affect other signaling pathways in different cancers or cell lines, like JAK/STAT in cholangiocarcinoma cells [14] or phosphoinositide 3-kinase (PI3K) and mitogen-activated protein (MAP) kinases in B16F10 melanoma cells [15]. In view of the mentioned effects of quercetin on different molecular pathways, we evaluated EGFR signaling pathways as a possible participating mechanism of quercetin to reverse rat liver preneoplastic lesions.

Here, we show that quercetin reverses liver preneoplastic lesions in the MRHM, influences the expression of EGFR, and modulates other related signaling pathways through the induction of the phosphorylation of Src-1, STAT5, and Sp-1. And finally, quercetin induces the recovery in the expression of IGF-1B, which reflects a recuperation of a better status of the liver.

## 2. Materials and Methods

### 2.1. Materials

Primary antibodies used were EGFR-t (C2C3, GeneTex), EGFR-p (S.684.2, Thermo), Src (36D10, Cell Signaling), Src-p (GTX24816, Genetex), STAT5 (9310, Cell Signaling), STAT5-p (9359, Cell Signaling), Sp1 (GTX110593, Genetex), Sp1-p (phosphor Thr739, Genetex), IGF1 (sc-9013, Santa Cruz Biotechnology), and *β*-actin (mouse monoclonal, CINVESTAV). Secondary antibodies used were goat anti-rabbit HRP (catalog number 62-6120, Invitrogen) or goat anti-mouse HRP (catalog number A9044, Sigma), WesternSure Chemiluminescent Western blotting reagent, and the Li-COR C-DiGit Blot Scanner (LI-COR Biosciences, Finland).

### 2.2. Animals and Treatments

Three groups of seven Fischer 344 male rats were used to perform this study. All the experiments were done according to the guidelines of the Institutional Committee for Animal Care and Use. Male rats, with 180 to 200 g of weight, were provided by the Unit of Production and Experimentation on Animals of Laboratory (UPEAL), at CINVESTAV, Mexico City, Mexico. All the rats were maintained under controlled temperature conditions with 12 hours light/dark cycles. Access to food and water was given ad libitum. Two groups of rats were subject to the MRHM, and an additional group was used as a control. In this last group, only the vehicles were administrated and were named the nontreated group (NT).

**Figure 1:**
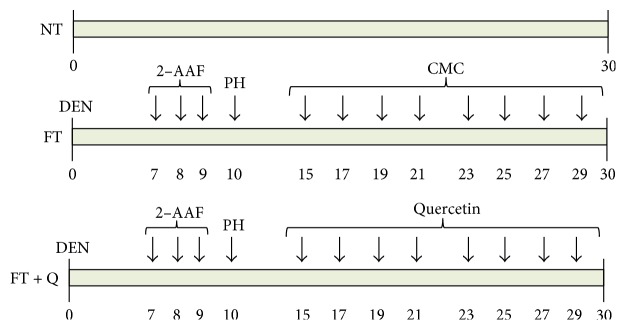
Groups of treatment. The rats were sacrificed 30 days after initiating the carcinogenesis treatment. NT, rats without treatment and just received the quercetin vehicle. FT, rats treated with MRHM and received the quercetin vehicle. FT + Q, rats treated with MRHM and received the quercetin. *n* = 7 rats per group.

In the two groups where the MRHM was used, an intraperitoneal single dose of 200 mg/kg of diethylnitrosamine (DEN) was administered in order to initiate a carcinogenic process. After this, three consecutive intragastric doses of 20 mg/kg of the promoter agent 2-acetylaminofluorene (2-AAF) were administrated in days seven, eight, and nine of the experimentation. Finally, ten days after initiation, a proliferative stimulus was induced through a partial hepatectomy including the 75% of the liver. From day fifteen until the sacrifice, quercetin in doses of 10 mg/kg was administrated every two days in one of the groups (FT + Q group). In the second group, only the vehicle for quercetin, carboxymethylcellulose (CMC) at 0.5%, was given (FT). The animals of the three groups were sacrificed at day thirty after DEN administration (Figure 1).

After sacrifice, livers were removed and each one was sectioned in two parts. One of them was frozen, cryosections of 20 *μ* of thickness were obtained, and slices were kept frozen until the GGT detection was performed. The other part was cryopreserved with liquid nitrogen for further protein analysis.

### 2.3. Histochemistry for Gamma-Glutamyl Transpeptidase (GGT)

Slices of 20 *μ* thickness on slides with gelatine were fixed in ethanol at −20°C for 10 minutes. Then, a Tris buffer solution (pH 7, containing gamma glutamyl-4-methoxy-2 naphthylamide (GMNA), glycyl-glycine, and fast blue) was added. Slices were incubated. After the incubation, the red color produced by a 100 mM solution of copper sulfate (CuSO_4_) made the enzymatic activity evident (all the chemicals were from Sigma Chemicals Co., St. Louis, MO). Using a polychromatic camera coupled to an OLYMPUS SZ045 microscope, images of the stained liver were captured.

### 2.4. Western Blot

From each rat of the three groups, total protein was obtained from a frozen liver sample of 100 mg of tissue. Tissues were mechanically homogenized in 1 ml of cold RIPA buffer supplemented with the protease inhibitors PhosSTOP and Complete (Roche Life Science, both). Extracts were centrifuged at 3500 rpm, at 4°C for 15 min. The supernatant was transferred to a new tube and centrifuged at 12,000 ×g at 4°C for 10 min. The supernatant was recovered again, and the total protein was quantified by the modified method of Bradford. Proteins were boiled 5 min in Laemmli buffer 2x and immediately placed on ice. 60 *μ*g of each sample were separated by SDS-PAGE in concentrated acrylamide gels between 6% and 15% (according to the protein in analysis). Later, proteins were transferred to polyvinylidene fluoride (PVDF) membranes. Nonspecific binding was blocked with TBS-T containing 10% of nonfat dry milk at room temperature for 2 h. Membranes were then incubated at 4°C overnight, with their corresponding primary antibodies. Primary antibodies were EGFR-t, EGFR-p, Src, Src-p, STAT5, STAT5-p, Sp1, Sp1-p, IGF1, and *β*-actin. After three washes, membranes were incubated with secondary antibodies goat anti-rabbit HRP and goat anti-mouse HRP at room temperature for 2 h. The membranes were newly washed, by three times, and the proteins of interest were visualized and analyzed using the WesternSure Chemiluminescent Western blotting reagent and the C-DiGit Blot Scanner (Li-COR. both).

### 2.5. Statistical Analysis

The number of GGT-positive foci and their area were quantified by using the analysis software AnalySIS Soft Imaging System GmbH. The intensity of the bands obtained in Western blot analysis was quantified with the software included in the C-DiGit Blot Scanner. The data were collected and statistically analyzed to generate the corresponding graphs, all this using the GraphPad Prism 4 software. The data were expressed as the mean ± SE.

## 3. Results

### 3.1. The Quercetin Reverses the Number and Area of Foci Related to Preneoplastic Lesions

**Figure 2:**
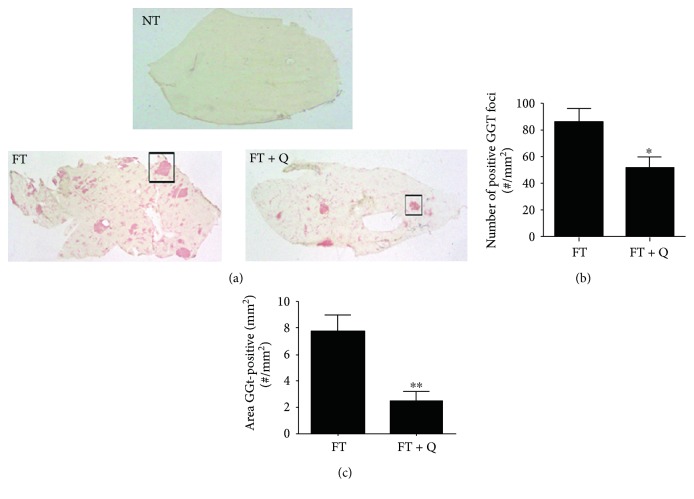
Effect of quercetin on GGT tumor-marker activity. (a) Preneoplastic lesions were detected by GGT activity (red area). (b) Number/mm^2^ of GGT-positive liver lesions. (c) Total GGT-positive area. A 2 mg/kg dose of quercetin showed reversion of preneoplastic lesions. ^∗∗^*p* < 0.01 and ^∗^*p* < 0.05

We use the modified resistant hepatocyte model (MRHM), in order to induce preneoplastic lesions in the liver of rats (FT group) and to evaluate the capacity of quercetin for reversing the lesions (FT + Q group). Briefly, lesions were induced with a single dose of DEN administrated to rats, whose characteristics have been previously described, to initiate the carcinogenic process. Seven days after DEN administration, the rats were treated during four days with the promoter agent 2-AAF, and a partial hepatectomy was done 10 days after DEN administration. From the fifteenth to the thirtieth day, one dose of quercetin (Q) was administrated to the rats in the FT + Q group every two days or only to the vehicle in the CMC group. All the rats, in the experimental and control groups, were sacrificed thirty days after the initiation (Figure 1). The expression pattern of the tumor marker GGT was analyzed to evaluate the effect of quercetin in chemoprevention of preneoplastic lesions. Gamma-glutamyl transpeptidase (GGT) is an enzymatic protein highly expressed in most models of hepatic cancer and is consider an early tumor marker on them. The activity of this enzyme was evaluated on liver slices, and the number of foci and their areas were determined. As it was expected, the activity of this enzyme could be detected in the liver of rats in the FT group (Figure 2(a), FT) but not in those of the control group without any treatment (Figure 2(a), NT). The FT and FT + Q groups present preneoplastic lesions as shown by GGT enzyme-positive staining, but preneoplastic lesions did not have a particular assigned distribution; they were in random distribution throughout the lobes. The observed difference was in the number and area of the lesions; the FT group was the one with more and with the most positive mark for GGT (Figure 2(a)). In general, on haematoxylin and eosin staining (data not shown), hepatocytes were observed altered, intensely basophilic, and translucent with vesicular nuclei and with prominent nucleoli, as expected. The treatment with quercetin, after the induction of premalignant lesions, produced a reduction in the number of foci positive for GGT (Figure 2(a), FT + Q). The quantitation of the number of foci positive for GGT indicates that there was a reduction of 58% in the number of preneoplastic lesions (Figure 2(b)). A reduction of the area in 81% of the preneoplastic lesions was observed; this decrease in number and area of preneoplastic lesions was statistically significant (Figure 2(c)). This result indicates that quercetin not only prevents the development of lesions as previously reported but also reverses them.

### 3.2. Diminished Expression of EGFR Was Observed When Premalignant Lesions Were Reversed by Quercetin

**Figure 3:**
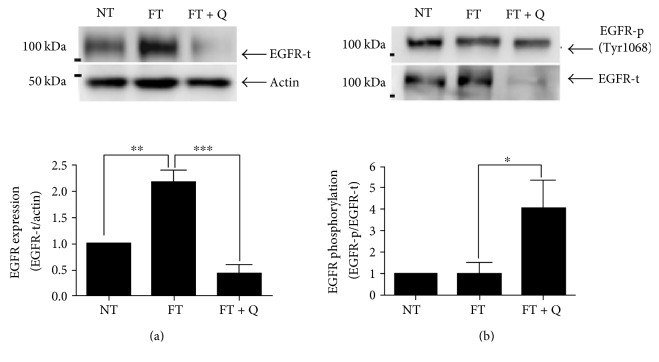
Expression of EGFR during quercetin treatment. (a) Western blot of EGFR expression. (b) Western blot of EGFR-p expression. A representative image and the analyses of seven independent events are shown. ^∗∗∗^*p* < 0.001, ^∗∗^*p* < 0.01 and ^∗^*p* < 0.05.

Because some signaling pathways are activated by quercetin through the EGFR protein, we analyze if EGFR participates in the observed effect of the quercetin reversion of preneoplastic lesions. The analysis by Western blot indicates that the total EGFR was 2.2 times overexpressed with respect to control values in the liver tissue of rats when preneoplasic lesions were induced (Figure 3(a), FT), but this was diminished to half of control values when quercetin was used to reverse the lesions. (Figure 3(a), FT + Q). To know the activation status of EGFR, the phosphorylated form of this receptor was evaluated by Western blot (Figure 3(b)). The activation of EGFR as shown by the ratio of phosphorylated EGFR versus total EGFR in the liver of rats with preneoplastic lesions was similar to that observed in the liver of rats without lesions (Figure 3(b), FT and NT); this ratio increases 3.0 times showing the activation of EGFR when the treatment with quercetin was used (Figure 3(b), FT + Q).

### 3.3. The Reversion of Premalignant Lesions by Quercetin Corresponds with the Activation of the STAT5 Signaling Pathway

**Figure 4:**
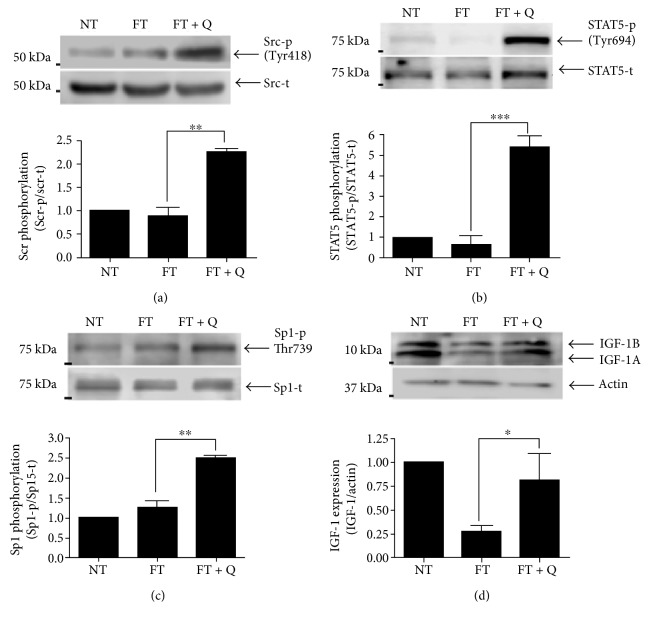
Signaling pathway activation through EGFR phosphorylation. (a) Western blot of phospho-Src. (b) Western blot of phospho-STAT5. (c) Western blot of phospho-Sp1. (d) Western blot of IGF-1A and IGF-1B. A representative image and the analyses of seven independent events are shown. ^∗∗∗^*p* < 0.001, ^∗∗^*p* < 0.01 and ^∗^*p* < 0.05.

The phosphorylation of EGFR induces the expression of several molecules through the activation of some signaling pathways; Src is one protein activated by EGFR, and it was increased in livers where quercetin was used to revert the preneoplastic lesions. The increase in the activation was 2.3 times more in the FT + Q group than in the FT group, which is statistically significant as could be observed by densitometric analysis. The activation of Src (Src-t) was similar in both the NT and FT groups (Figure 4(a)). In summary, the activity of Src was increased during the reversion of lesions, when quercetin was used.

The evaluation of some signaling pathways activated by EGFR allowed us to observe an increase in the activation status of STAT5. STAT5 was phosphorylated in a similar way in the liver from rats in the NT and FT groups. But an important increase in the activation of STAT5 (STAT5-p) was observed in the group where the preneoplastic lesions were diminished by the treatment with quercetin (4.4 times more than that in the NT group). The higher activation status of STAT5 in the FT + Q group, with respect to that observed in the FT group, was also statistically significant (Figure 4(b)). So, we could think that quercetin induces the phosphorylation of STAT5, increasing its activity.

The activation of Sp1 along with STAT5 has been previously reported. Then, the activation status of Sp1 was also evaluated in the liver of rats in the study. The analysis of the group where the treatment with quercetin was used to revert preneoplastic lesion revealed an increase in the activation of Sp1which was quantified by densitometry. The increase observed was more than 1.4 times with respect to that in the FT group (Figure 4(c)). There was no significant difference in the activation status of Sp1 between the NT group and FT group. In such manner, Sp1 is activated along with STAT5 when quercetin is used.

The target gene IGF-1 that is an indicator of the functional status of the liver was also evaluated. As we expected, the induction of preneoplastic lesions by using the MRHM resulted in a 74% decrease of IGF-1 expression (Figure 4(d), FT). When we analyzed its expression in the liver of rats in the FT + Q group, the expression of IGF was recuperated, from 26% to 83% of control values (Figure 4(d), FT + Q group). This last result is a reflection of the functional status of the liver in the three groups.

## 4. Discussion

The ability of quercetin to reverse preneoplastic lesions in the liver was tested in the MRHM. This model has been used by us [9] and other authors in order to observe the three different stages associated with the development of HCC: initiation, promotion, and progression [11, 16, 17]. The evaluation of the presence of preneoplastic lesions was done through the activity of the GGT. This enzyme has been analyzed in the different stages, and it is expressed all along from the initiation to progression in models of chemical carcinogenesis and led to the identification of preneoplastic cells [18, 19]. The GGT enzyme is a focal marker commonly used and also considered as a tumor marker. We found that the administration of quercetin reverse the preneoplastic lesions in this model, which was evident by the lower number of altered hepatic foci (positive for GGT) but the major effect was seen in the decrease of the area. In the FT and FT + Q groups, we observed lesions with GGT staining; however, it is evident that in the FT group, multiple lesions were observed and the FT + Q group showed a drastic effect on GGT decline. In the quantification of GGT, 0.01 mm^2^ was a low-end measure to discard stains not associated with preneoplastic lesions (ductile cells) with a preferential circular shape. Several reports indicate that quercetin has diverse antitumor activities in different cancers or precancerous lesions. And its activity occurs through the regulation of different signaling pathways like *β*-catenin/Tcf or AMP-activated protein kinase (AMPK) in colon cancer cells [20, 21], NF-*κ*B and AP-1/JNK in human hepatoma cells [22], or EGFR/PI3K/Akt pathway in prostate cancer [13] and EGFR in liver cells [12]. The signaling pathways are maintained overexpressed during the progression of cancer. So if quercetin is able to regulate several pathways during the progression, it was expected to cause a reversible effect over the lesions.

We observed a clear decrease in the number of altered hepatic foci and their area, which coincides with the diminished expression of EGFR and then a lower quantity of phosphorylated EGFR. But due to the increase in the ratio of phosphorylated EGFR, we evaluated the activation of Src and we found that phosphorylated Src was higher in the tissues treated with quercetin. This last result agrees with previous reports which indicate that the transactivation of EGFR can occur through GPCR ligands and some cytokines. These can induce phosphorylation of EGFR in the absence of a ligand and, as a consequence, the activation of Src or Janus tyrosine kinase 1 (Jak1) [23]. In addition, Src can activate different STATs. STATs could be activated by JAK kinases, growth factor receptors (EGFR), or Src family members [24]. Here, we showed that STAT5 is phosphorylated in the liver of rats treated with quercetin to reverse the preneoplastic lesions. The activation of STAT5 by Src has been previously reported [24]. Even more, in rat hepatocytes in primary culture, it has been proved that EGF induces the activation of STAT5 apparently through a Src-dependent mechanism [25]. Moreover, Src induces the expression of proteins, like MMP2, through the ERK/Sp1 pathway [26]. And a signaling interaction between STAT5 and Sp1 has also been proved, mainly in the expression of cyclin D2 [27]. Here, we found that Sp1 is more active in the liver of rats treated with quercetin. It is important to highlight that Src, STAT5, and Sp1 were more phosphorylated in the liver of rats when treated with quercetin. But there were no significant differences in the phosphorylation of Src, STAT5, and Sp1, between the groups with induced preneoplastic lesions and controls. Therefore, we can deduce that the reversion of preneoplastic lesions by quercetin occurs by a direct effect on signaling pathways responsible for the development of lesions, like EGFR, and additionally to the activation of signaling pathways whose molecules are considered as a tumor suppressor, like STAT5 [28]. The STAT5 activity as a tumor suppressor is attributed mainly to the control of reactive oxygen species through the expression of PUMA, BIM, and NOX4 [28]. We also analyzed the expression of IGF-1, and it was diminished in the liver of rats with preneoplastic lesions. This was expected since, previously, it has been showed that diminished expression of IGF-1 is related to the progression of different liver diseases [29]. Specifically, in HCC, serum levels of IGF-1 are diminished and it has been associated with poor prognosis [30, 31], but the patients with the highest levels of IGF previously to antiangiogenic treatment are associated with a better disease control rate [31]. Our results showed that the treatment with quercetin recuperates IGF-1 almost to control values. Even if the level did not reach that in the control group, this increase in the expression of IGF-1 coincided with the lower number and area of the neoplastic lesions. This allowed us to conclude that this last reflects a better control of the preneoplastic lesions in the liver of rats treated with quercetin.

## 5. Conclusions

In conclusion, we showed that besides the known effect of quercetin as a protective agent, quercetin treatment is also useful to reverse preneoplastic lesions. This effect is observed not only on the decreased proliferation, but we also cannot discard the possibility that tumor-initiating cells were removed. Therefore, the expression of EGFR as well as the modulation of its activation state participates in the decrease of the preneoplastic lesions. Also, the activation state of Src, STAT5, and Sp1 as the basal IGF-1 restoration participates on the decrease in the number and size of preneoplastic lesions caused by quercetin.

## Abbreviations

HCC: Hepatocellular carcinoma
MRHM: Modified resistant hepatocyte model
DEN: N-Diethylnitrosamine
2-AAF: 2-Acetylaminofluorene
GGT: Gamma-glutamyl transpeptidase
EGFR: Epidermal growth factor receptor
PI3K: Phosphoinositide 3-kinase
MAP: Mitogen-activated protein
NT: Nontreated group
Jak1: Janus tyrosine kinase 1.
